# Trochleoplasty in major trochlear dysplasia: current concepts

**DOI:** 10.1186/1758-2555-4-7

**Published:** 2012-02-21

**Authors:** Philippe Beaufils, Mathieu Thaunat, Nicolas Pujol, Sven Scheffler, Roberto Rossi, Mike Carmont

**Affiliations:** 1 Orthopaedic Surgery Department, Centre Hospitalier de Versailles, Versailles, France; 2 Versailles Saint Quentin University, Versailles, France; 3 Chirurgisch, Orthopädischer PraxisVerbund, Sports Medicine & Arthroscopy Service, Berlin, Germany; 4 Department of Orthopaedics and Traumatology, University of Torino, Turin, Italy; 5 Princess Royal Hospital, Shropshire & Sheffield University Teaching Hospitals NHS Foundation Trust, Sheffield, UK

**Keywords:** Trochlea, Patello femoral dysplasia, Patellar instability, Trochleoplasty

## Abstract

Trochleoplasty is the theoretical solution to persistent symptoms (pain and/or instability) related to trochlear dysplasia where there is not only a trochlear flatness but also a trochlear prominence. The threshold of prominence indicating surgical intervention has as yet not been determined. A bump of 5 mm is generally accepted as the inferior limit. Given the interventional nature of this demanding procedure, it should be proposed in selected cases after considerable discussion with the patient. Trochleoplasty is indicated as a primary procedure for major trochlear dysplasia with a prominence > 5 mm. Stabilization is obtained in most of the cases with the risk of residual mild anterior knee pain. It is also indicated as a salvage procedure when a previous surgery failed. Despite the reputation of the procedure, the published results are encouraging in terms of prevention of re-dislocation, satisfaction index, and radiological outcomes. Post-operative stiffness is the main complication, which may require manipulation under anaesthesia or arthroscopic arthrolysis. There are few other complications reported and to date secondary necrosis of the trochlea has not been reported. Technically speaking, the deepening trochleoplasty is a difficult procedure without reliable landmarks. We propose a recession wedge trochleoplasty which is an easier procedure. It is never undertaken as an isolated procedure, but always in conjunction with other realignment procedures of the extensor apparatus according to the "a la carte" surgery concept.

## Introduction

The importance of a dysplastic trochlea as a component of patellar instability (especially recurrent dislocation or habitual dislocation) has been recognized for many years. It is usually combined with other static or dynamic abnormalities such as genu recurvatum, patella alta, patellar tilt, increased Q angle and bone torsional abnormalities.

Major trochlear dysplasia is characterized by the combination of a flat and/or prominent trochlea proud of the anterior femoral cortex which offer inadequate tracking during flexion and lead to patella subluxation respetively [1,2].

Many surgical techniques have been proposed for the treatment of patellar instability. Trochleoplasty has been described as corrective treatment for bony abnormalities for many years with the goal of restoring normal anatomy. Correcting the trochlear depth abnormality plays a major role to stabilising the patella because it facilitates proper entrance of the patella into the groove of the trochlea. In our experience the restoration of the trochlea groove by trochleoplasty prevents future patellar dislocation and is effective in reducing anterior knee pain.

Elevation of the lateral trochlear facet was first described by Albee [3] in 1915, followed by deepening trochleoplasty [2,4-12] which tries to create a new sulcus by removing subchondral bone. Recently Goutallier [13] proposed an easier concept, termed Recession Trochleoplasty, in which the bump is solely corrected with the trochlea remaining flat. This has now been adopted as our preferred technique [14].

Trochleoplasty is considered as a demanding technique and frequently may be avoided due to a lack of familiarity. However it can be a useful addition to the surgical armamentarium of the patellofemoral surgeon and has precise indications.

Trochleoplasty can be proposed as a primary procedure for primary trochlea dysplasia or as a salvage procedure [13] in case of failure after previous patellar alignment surgery, principally Anterior Tibial Tubercle Transfer (ATTT).

In the large majority of the cases, trochleoplasty is performed in association with other procedures (bony procedures such as ATTT transfer, or soft tissue procedure such as medial patello femoral ligament (MPFL) reconstruction). This combined procedures follow the concept of "à la carte" surgery described by Henri and David Dejour [1,7], which tries to address all the abnormalities during one surgical intervention.

### Principles

**Figure 1:**
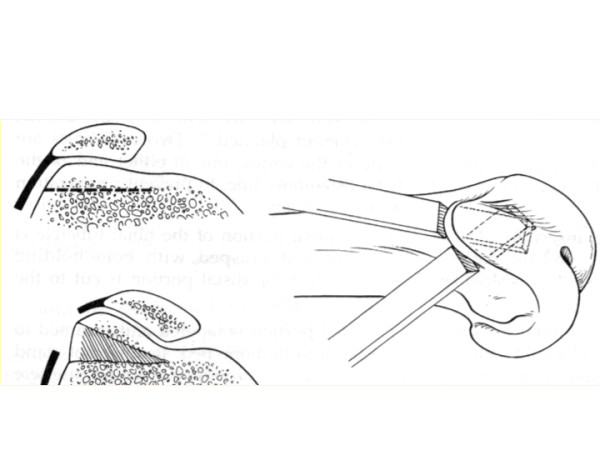
**Elevation of the lateral facet, according to the Albee technique**.

The first trochleoplasty performed was the elevation of the lateral trochlea facet described by Albee [3] (Figure 1). This addressed a flat trochlea by increasing the trochlear prominence. This method is now generally considered to be erroneous as it increases the patellar constraints, leading to secondary osteoarthritis and as a result lateral trochlear elevation has fallen out of favour.

**Figure 2:**
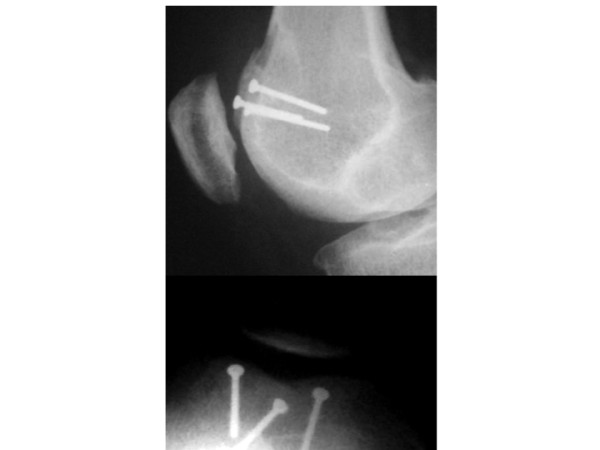
**Deepening throchleoplasty**. The trochlear groove has been restored but note the incongruency between the flat patella and the deepened trochlea.

The second method is the deepening trochleoplasty. In 1966, operations to correct the abnormality by deepening the sulcus were introduced by Masse [4]. He suggested the removal of subchondral bone and to impact the articular cartilage with a punch to recreate a central sulcus. This technique was later modified by Henri Dejour [2] who performed an osteotomy of both femoral condyles to create a V-shaped trochlear groove. Von Knoch et al. [5] described another technique known as "the Bereiter technique", in which an osteochondral flap is raised from the trochlea and a bony sulcus is fashioned using burrs. The flaps are then depressed making a smooth groove and fixed by vicryl tape. This technique has been later described under arthroscopic control by Blønd and Schottle [6]. Deepening trochleoplasty by whichever method, is logical because it reduces the flatness and the prominence and tries to restore a normal anatomy (Figure 2). There are several key points to be considered whist performing deepening surgery: where to locate the trochlea sulcus, when the trochlea is flat?

• What about the congruency between a flat dysplastic patella on a deepened trochlea? (Figure 2)

• What is the morbidity of this demanding technique, particularly bone healing and the risk of subchondral bone or cartilaginous necrosis?

**Figure 3:**
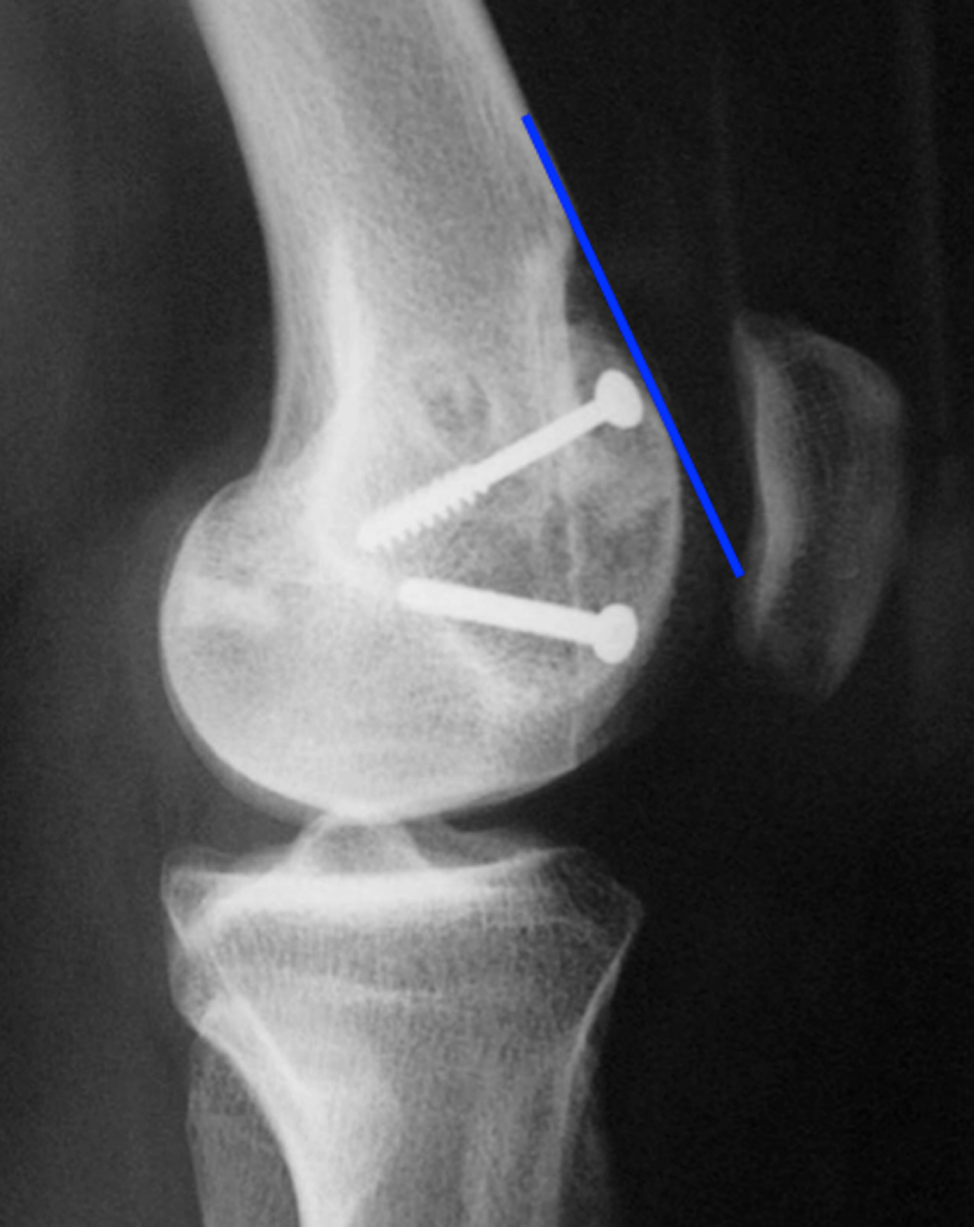
**Recession trochleoplasty**. There is a reduction of the prominence but the flat trochlea remains (crossing sign).

The third type of trochleoplasty has been described by Goutallier et al. [13] who performed a "recession"-type trochleoplasty. Here the prominent dome shaped anterior surface of the distal femur is recessed to the level of the anterior femoral cortex without deepening the groove itself. The aim is not to fashion a groove but to reduce the prominent bump without modifying the patellofemoral congruence. This procedure is technically less demanding than a deepening trochleoplasty (Figure 3). Recession trochleoplasty has several advantages: diminishing the trochlear bump improves patellar tracking, reduces lateral subluxation and decreases patellofemoral constraint by increasing the angle between the quadriceps muscle force and the patellar tendon force. This has now become our preferred technique and we have reported the outcome of 24 cases of recession trochleoplasty performed between 2004 and 2009 [14] (mean age: 25; 12 primary procedures and 8 salvage procedures). Recession Trochleoplasty was always performed together with another additional procedure: 16 ATT tranfers, 8 MPFL reconstructions.

### Pre-operative imaging

Pre-operative imaging forms the key to determine when trochleoplasty is indicated. We have established a standard protocol of plain radiographs for visualisation of the patello-femoral joint. These consist of AP view, lateral view at 20° of flexion, lateral view in full extension with quadriceps contraction, skyline views at 30° in neutral rotation of the leg [15] and in external rotation (in order to demonstrate an eventual lateral subluxation). Additional bone imaging is provided by computed tomography [16].

**Figure 4:**
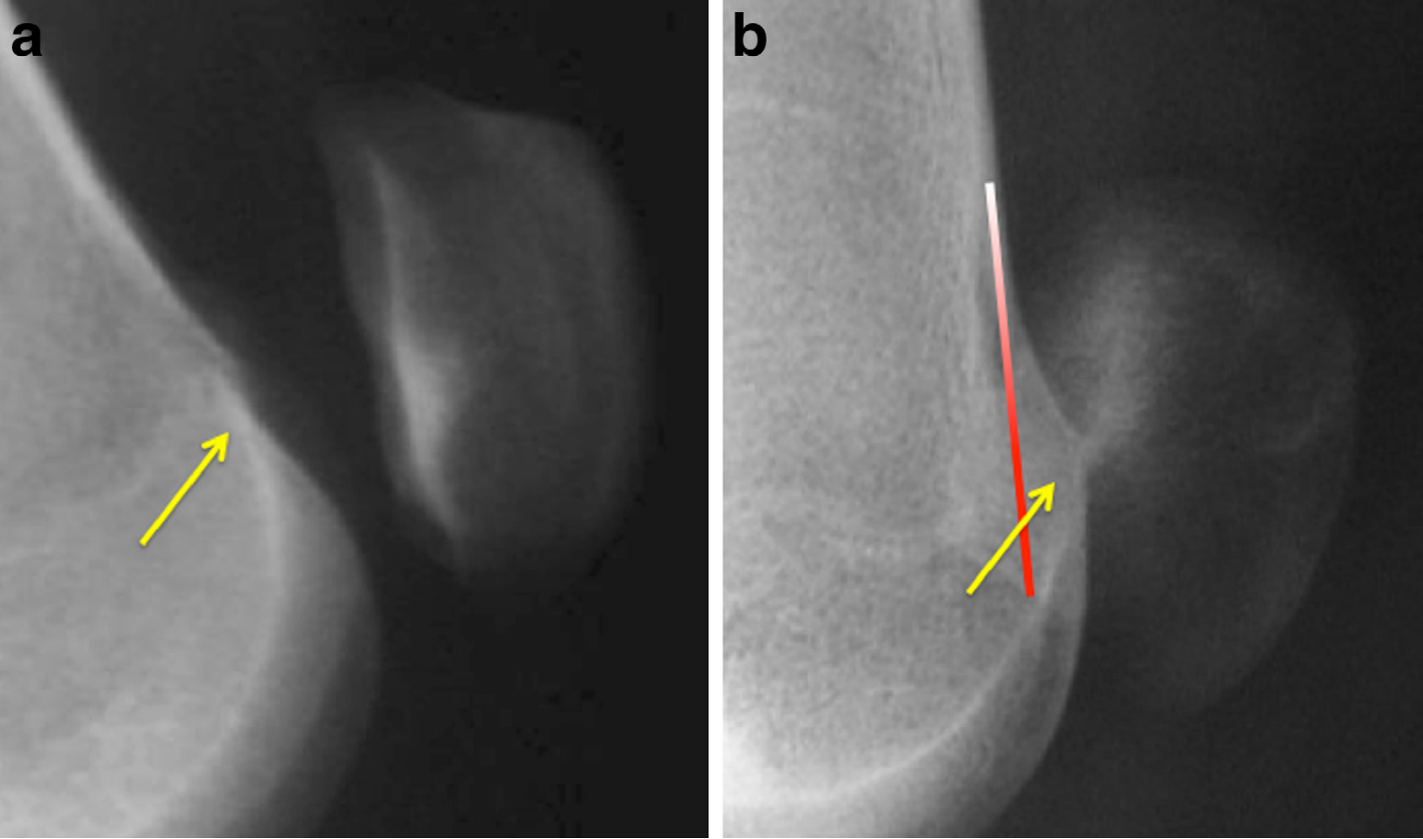
**Different types of trochlear dysplasia a: crossing sign without prominence b: crossing sign and marked prominence demonstrating a major dysplasia**.

The projection of the lateral radiograph is critical. By ensuring that the posterior aspect of both the medial and lateral femoral condyles are superimposed the bony anatomy of the trochlea can be compared. A number of key measurements and lines have been described based upon this true lateral projection [1]:

- the Crossing Sign described by Walch characterizes the trochlea flatness.

- the trochlear bump or prominence is measured by the distance between a line tangential to the anterior femoral cortex, and a line parallel to this through the trochlear groove. A bump > 5 mm characterizes a major dysplasia (Figure 4)

- Patellar height may also be determined to consider an ATTT distalization procedure. We prefer to use the Caton Deschamps [17] index > 1.2

**Figure 5:**
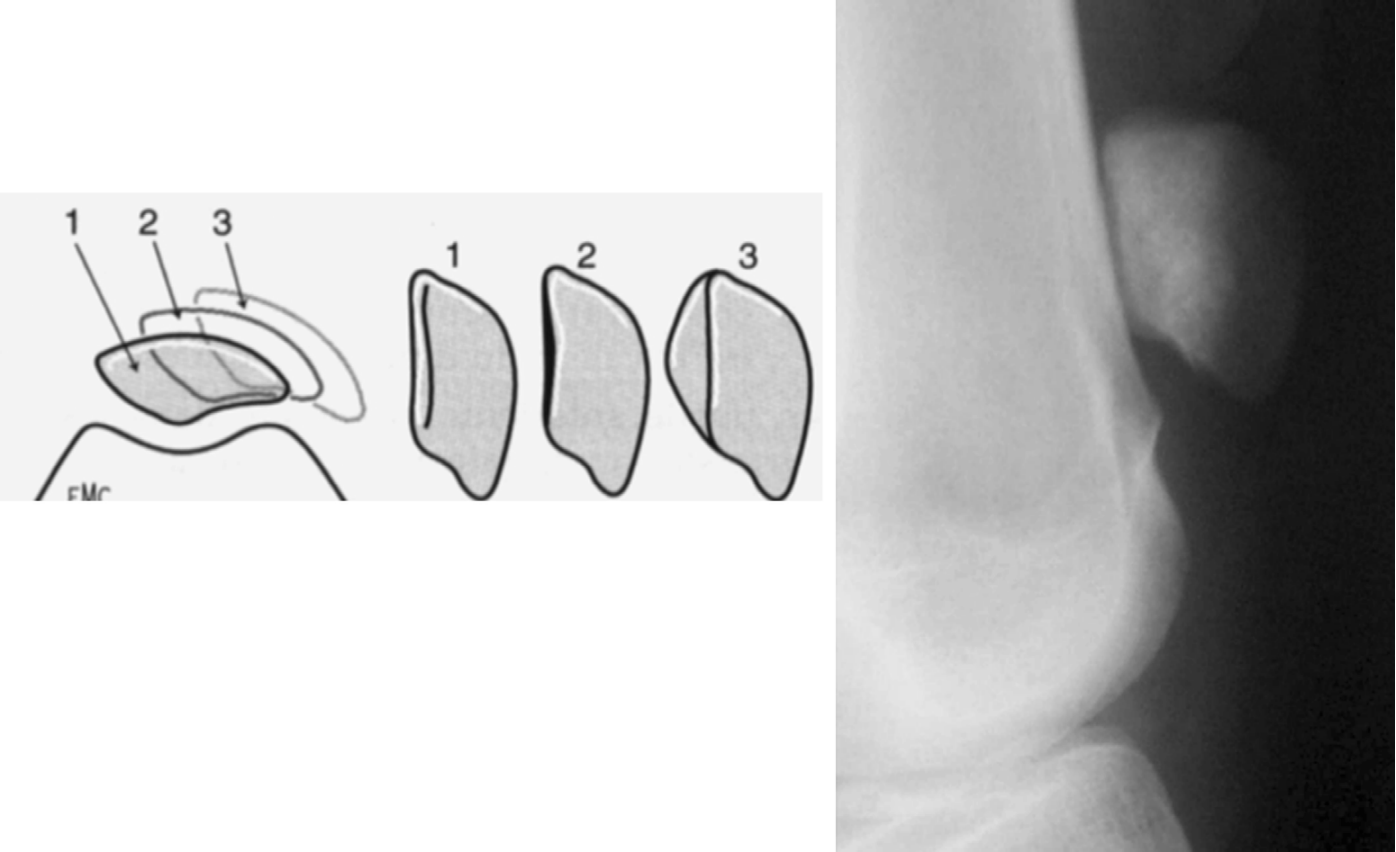
**lateral view in complete extension and quadriceps contraction**. It allows to assess patellar tilt, according to the shape of the patella.

Lateral view in complete extension with quadriceps contraction allows assessment of patellar tilt. (Figure 5) The "thick patella sign" characterises a tilted patella, which appears thickened front to back.

**Figure 6:**
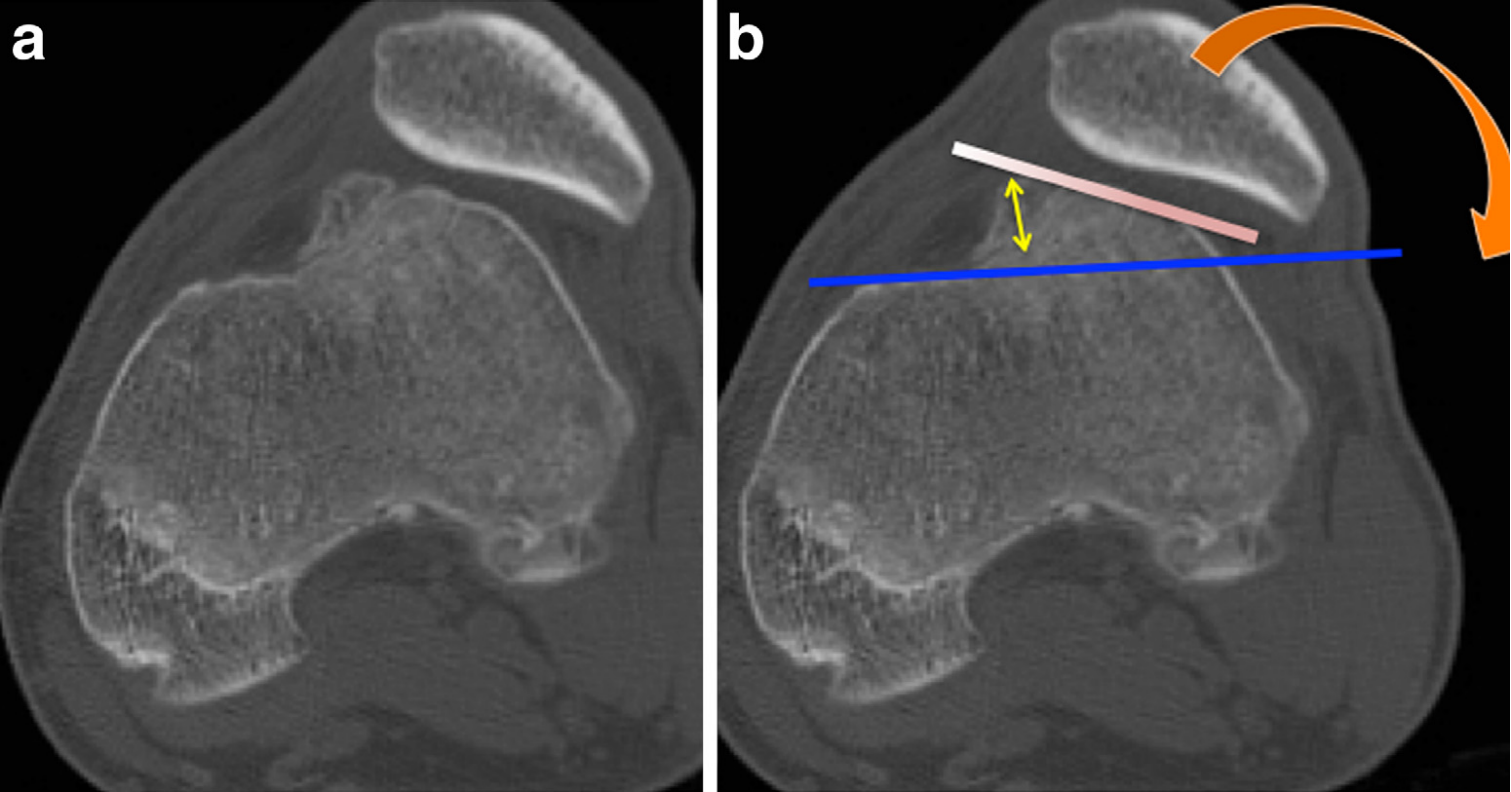
**CT scan**. It directly evaluates the trochlear prominence. The orientation of the trochlear plane and the trochlear lateralization explain patellar tilt and subluxation.

CT scanning confirms the trochlear flatness and the trochlear prominence on sagittal sections, which can also be measured (Figure 6) according to David Dejour's classification [7]. It is important to consider that the dysplastic trochlea is lateralized compared to the center of the femoral epiphysis. This lateralization must be taken into account during trochleoplasty procedure. The CT scan also measures the distance between tibial tubercle and the trochlear groove (TTTG). This is the traditional image based determination of an increased Q angle [16]. Finally CT scan also permits the assessment of the patellar tilt in extension: a tilt of more than 20° may be considered as an indication for additional soft tissue reconstruction.

### Operative technique

The procedure is performed supine. A tourniquet minimises bleeding from the exposed areas of cancellous bone. Arthroscopy may be performed to confirm the absence of cartilage defect prior to trochleoplasty surgery. The differing techniques are described as follows:

1 Deepening Trochleoplasty

**Figure 7:**
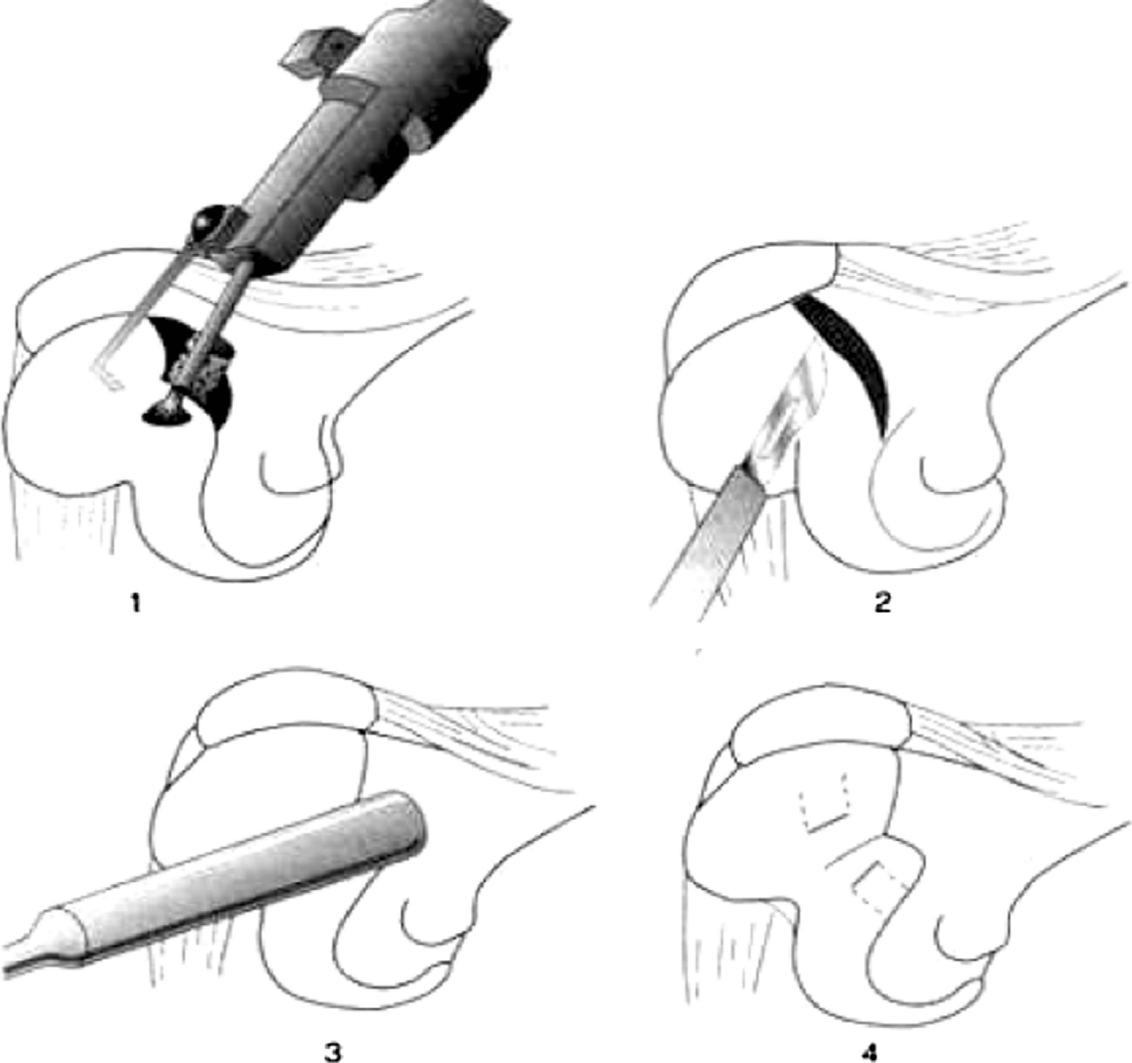
**The four steps of deepening trochleoplasty**.

David Dejour [18] proposes the following technique for deepening trochleoplasty (Figure 7). Arthrotomy is performed through a mid vastus medial approach. The patella is translated laterally without eversion. Peritrochlear tissue are then excised in order to visualize the anterior femoral cortex, to define the amount of bone to be removed. The new trochlear sulcus is then drawn starting from the top of the intercondylar notch and directing proximally with a 3 to 6° of valgus. Lateral and medial facets are also demarcated. To access the under surface of the trochlea, a thin strip of cortical bone is removed from the osteochondral edge. Cancellous bone is removed from the undersurface of the trochlea. A drill with a depth guide of 5 mm is used to ensure uniform thickness of the osteochondral flap, this maintains an adequate amount of bone beneath the trochlear articular cartilage. The produced shell must be thin enough to be modelled without being sustaining a fracture. More bone is removed from the central portion at the location of the new sulcus. The groove and sometimes the medial and lateral margin must be osteotomized. The osteochondral flap is then replaced and moulded by gentle tapping with a punch. The new trochlea is then fixed with two small staples (1 mm in diameter), one in each side of the groove. One arm is fixed in the upper part of the trochlear cartilage, the other one in the anterior femoral cortex. The staple is sunk deep to the superior surface of the cartilage. Patellar tracking is tested by flexing and extending the knee.

2 Recession trochleoplasty

**Figure 8:**
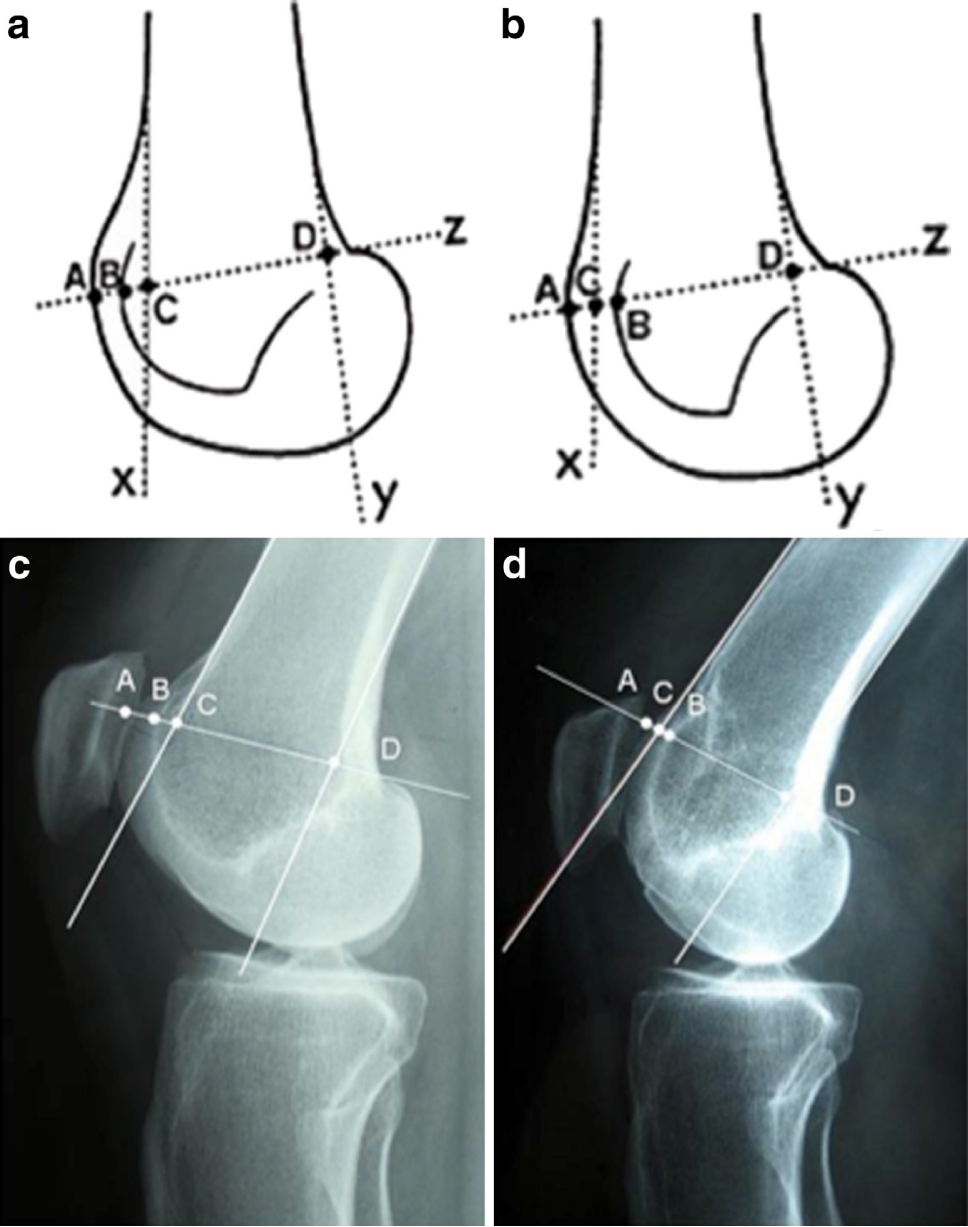
**Pre and Post-operative bump height measurement technique**. A and B: Drawings showing the Dejour and Walch method for calculating the bump height. Point "D" is the junction between the posterior cortex and articular cartilage. Bump height is measured between points "B" and "C". C: Pre operative lateral radiograph: the boss height is positive. D: Post operative lateral radiograph: the boss height is now negative.

**Figure 9:**
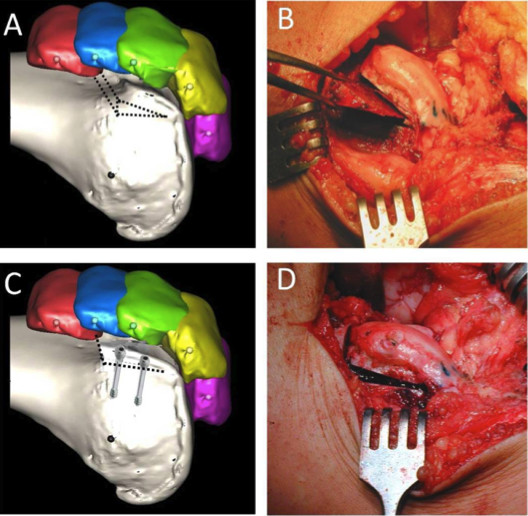
**The recession wedge trochleoplasty surgical technique**. A and B: The base of the wedge which is removed should be the same in millimeter that the value of the trochlear bump in order to allow the trochlea to settle into a deeper position, without modifying the trochlear groove. C and D: The correction is obtained after removal of the proximally based wedge by progressively applying a pressure on the trochlea. Fixation is carried out with two 3.5 mm cancellous screws, positionned just laterally to the cartilage surface.

**Figure 10:**
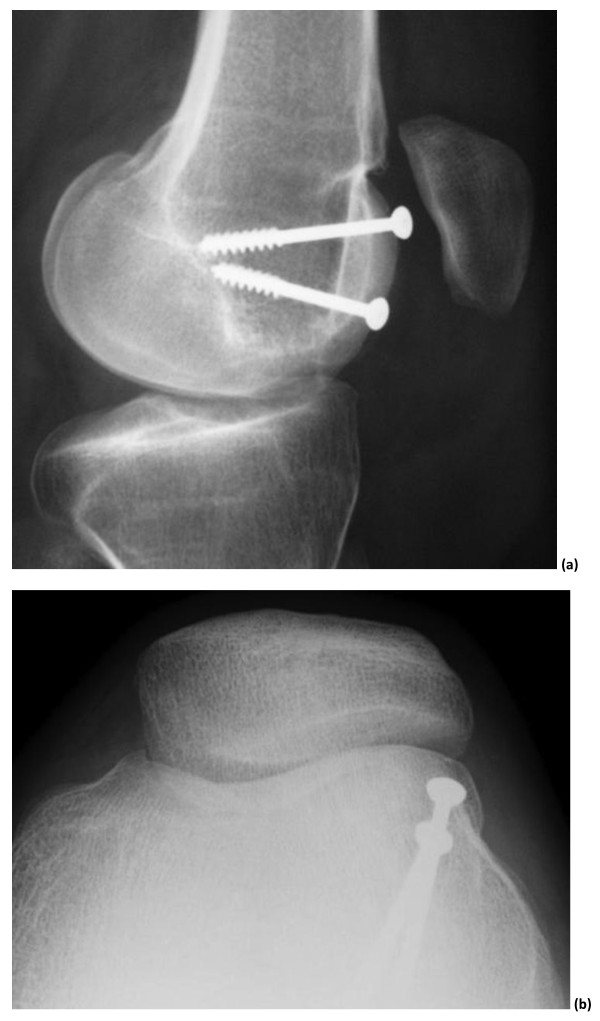
**Post operative X rays after Recession wedge trochleoplasty**. A: lateral view showing the reduction of the trochlear porminence. B 30° patello femoral view showing the extracartilaginous position of the screws.

We prefer to perform a lateral approach as the dysplastic trochlea lies on the lateral aspect of the femoral epiphysis. The incision is made just lateral to the patella, extending from the superior pole to the level of patella to beyond the tibial tubercle onto the anterior ridge of the tibia. This permits a tibial tubercle transfer to be performed during the same procedure if required. Our technique aims to treat the underlying anatomical abnormality without compromising the articular surface. Once the lateral retinaculum is exposed, a lateral arthrotomy is performed using a size ten blade. The synovium is excised and tethering scar tissues proximally and distally are released. The size of the wedge to be excised and the angle to be corrected are guided by pre-operative imaging and measured intra-operatively (Figure 8). The osteotomies are initially drawn on the bone with a dermographic pen according to the pre-operative planning (Figure 9). Using a reciprocal saw, the antero-posterior cut is performed first, 5 mm above the trochlea. Then the posterior cut is made, parallel to the frontal plane of the femur, from the lateral side, and directed medially. It is more precise to start the cut with a rigid osteotome and to complete it with the saw. The distal extent of the osteotomy should be approximately 5 mm away from the sulcus terminalis in order to give an optimal distal osteochondral hinge and to allow closing the wedge easily. Then the anterior oblique osteotomy completes the bone cuts linking the first two cuts. The proximal based bone wedge is then removed and correction is achieved by progressively applying sustained gentle digital pressure on the trochlea. The amount of bone removed is just enough to allow the trochlea to settle into a deeper position, without modifying the trochlear groove. The correction is secured using 3.5 mm cancellous screws, positioned just laterally to the cartilage surface (Figure 10). We now use two lateral screws only and so far have had no problems.

Post operatively the knee is placed in an extension brace for the initial three weeks. Full weight bearing is allowed. Knee flexion is restricted to 0 to 60 degrees for the first three postoperative weeks, and then slowly increased to reach 90 degrees on the sixth week. Return to sports is allowed at 6 months.

## Results

### Complications-Safety

The risks of the deepening trochleoplasty include breaking of the osteochondral flap, distal detachment, and creating a too thin flap decreasing its blood supply. There are still concerns about the viability of the articular cartilage after trochleoplasty. The recession wedge trochleoplasty has a decreased the risk of chondral damage and necrosis. Since the dysplastic segment of trochlea is lifted as a single osteochondral block and there is no need to fashion a new groove by cutting the osteochondral flap, it is possible to preserve a greater amount of subchondral bone. This makes this the recession arthroplasty a more attractive option for older patients with less pliable cartilage with decreased risk of possible serious and irreversible articular and subchondral injury. In our series, we reported no cases of chondrolysis, subchondral necrosis or non-union of the osteochondral block.

It is worthy of note that in cases of recession trochleoplasty the wedge and the trochlear recess are flat and complementary, whereas in the deepening trochleoplasty, the osteochondral flap might not tally perfectly with the V shaped recipient bone bed. Any small areas of separation between the two surfaces could slow down the osteointegration process. Similarly the use of screw to stabilise osteotomy rather than sutures may increase compressions between the two surfaces. Surprisingly chondrolysis has never been reported with the deepening trochleoplasty.

Schottle [19] studied the cartilage viability after the Bereiter trochleoplasty He found that tissue in the trochlear groove remained viable, with retention of distinctive hyaline architecture and composition and only a few minor changes in the calcified layers.

Post op stiffness is of considerable concern [8,11-13] and varies from 2 to 46% (Table 1). In our series, one patient (with combined MPFL) required arthroscopic arthrolysis for knee stiffness one year after the index operation. Another patient required an arthroscopic supratrochlear exostosectomy for a persistent ridge responsible for pain. He was also satisfied and had no complaint at last follow up and reported no further episodes of instability.

**Table 1:** Literature review: trochleoplasty for major dysplastic trochlea

Authors	Year	N	F-U	Previous	Indication		Surgical technique	Additional	Results		Complications	
			**(months)**	**surgery (%)**	**Pain (%)**	**Instability (%)**		**procedures (%)**	**Satisfaction (%)**	**Failure (%)**	**Pain (%)**	**Stiffness (%)**

Masse et al [4]	1978	18	40	?	61	100	Deepening trochleoplasty	?	?	0	11	17

Reynaud et al [10]	1995	40	33	50	?	97	Dejour trochleoplasty	100	77	2	?	7

Gougeon et al [9]	1996	51	40	41	?	80	Albee(75%) deepening (25%)	?	?	2	?	11

Goutallier et al [13]	2002	12	48	92	100	0	Recession wedge trochleoplasty	64	67	0	83	0

Verdonk et al. [12]	2005	13	18	77	100	54	Dejour trochleoplasty	23	77	0	46	46

Von Knoch et al. [5]	2006	45	96	33	77	100	Bereiter trochleoplasty	?	100	0	91	0

Donell et al. [8]	2006	15	36	60	?	100	Modified Dejour trochleoplasty	100	80	0	?	33

Utting et al.[11]	2008	59	24	30	?	100	Bereiter trochleoplasty	49	92	0	15	2

Thaunat et al [14]	2011	20	34	40	60	100	Recession wedge trochleoplasty	100	94	10	100	5

*N *number of cases

*FU*follow-up

### Clinical outcomes

To date published outcomes of both deepening and recession trochleoplasty are similar with improved subjective outcome scores reported in the short term [4,8-14,18] (Table 1). Comparisons between series are difficult since the surgical procedures and follow-up are variable, the number of patients is often small and patients have been operated for mixed indications of pain rather than dislocation [12,13]. Moreover, it is not possible to assess the participation of trochleoplasty in the patellofemoral stability, because it is rarely solely performed and other abnormalities are corrected as part of the surgical procedure. As a result of this, there is a lack of high level studies reported in the literature.

Goutallier has reported a case series with 67% of patients reporting that they were either satisfied or very satisfied with the outcome of surgery where trochleoplasty was performed as a salvage procedure. Other series show 100% satisfaction rates (Table 1).

In our series, the operation failed to stabilize the patellofemoral joint in only two cases. The average objective knee score at last follow up was 80 (+/-17) for the Kujala score [20], 70 (+/-18) for the KOOS and 67 (+/-17) for the IKDC. Patients who had a previous surgery, and those with patellofemoral chondral lesions noted during the surgery or degenerative changes on the preoperative radiographs were noted to have a lower Kujala score at last follow up.

Interestingly all the patients operated for painfree instability (n = 7) reported have slight pain. This was located at the site of screws to reattach the tibial tubercle and so was not directly related to the trochleoplasty itself. All the patients with pre-operative pain bar one (n = 11) report significant pain improvement at last follow up.

### Radiological outcome

Both deepening or recession trochleoplasty are able to reduce the trochlea bump. In our series, the trochlear groove height changed from an average of 4.8 mm pre-operatively to an average of -0.8 mm post-operatively (Figure 7, 8, 10). Patellar tilt changed from an average of 14° (6° to 26°) preoperatively to an average of 6° (range -1° to 24°). It is interesting to note there was no significant difference in the correction of the patellar tilt angle when comparing the groups who had or not the adjunction of a MPFL reconstruction. Thus our series suggests that MPFL reconstruction is not be necessary when a recession wedge trochleoplasty is performed. The reduction of the boss height allows the avoidance of lateral misdirection and facilitates the sliding of the patellar into the trochlea recess.

Although the deepening or recessing trochleoplasty is effective in reducing anterior knee pain, it does not halt the progression of patellofemoral arthritis, although the follow up of the above studies is too short to draw any definitive conclusions. In our series [14], at the time of the latest follow-up, six knees had osteoarthritic changes in the patellofemoral compartment according to the classifcation by Iwano et al [21]. These are similar to the results obtained with deepening trochleoplasty [5]. Trochleoplasty cannot be proposed as a prevention of late osteoarthritis.

## Conclusion

Trochleoplasty is indicated as a primary procedure for major trochlear dysplasia with a prominence > 5 mm. Stabilization is obtained in most of the cases with the risk of residual mild anterior knee pain. Trochleoplasty can be also proposed as a salvage procedure when a previous surgery failed. In these cases, one can expect a stabilization of the knee and improvement of anterior knee pain.

Reported results are encouraging in terms the prevention of re-dislocation, satisfaction index, The rate of complications is low. Long terms outcomes have not been reported and there are no consistent data on the capacity to prevent secondary arthritis

Technically speaking, the deepening trochleoplasty is a difficult procedure. Recession wedge trochleoplasty is an easier procedure. It is never an isolated procedure but always in conjunction with other realignment procedures according to the "a la carte" surgery concept.

## Abbreviations

ATTT: Anterior tibial tubercle transfer; MPFL: Medial patello femoral ligament; TT-TG: distance between tibial tubercle and trochlear groove on CTscan.

## Competing interests

The authors declare that they have no competing interests.

## Authors' contributions

PB collected the data and wrote the manuscript/MT, NP collected the data and reviewed the manuscript/MC, SS, RR reviewed and approved the manuscript.

## Fundings

None.
